# Harmonizing Measurement of Satisfaction With Acne Scar Treatments: Can We All Sing the Same Tune?

**DOI:** 10.1097/DSS.0000000000003107

**Published:** 2021-06-09

**Authors:** Esther J. van Zuuren, Bernd W.M. Arents, Sofieke Vermeulen, Jerry Tan

**Affiliations:** * Department of Dermatology, Leiden University Medical Center, Leiden, the Netherlands; † Skin Patients Netherlands, Nieuwerkerk a/d IJssel, the Netherlands; ‡ Department of Dermatology, Reinier de Graaf Hospital, Delft, the Netherlands; § Department of Medicine, Western University, Windsor Campus, Ontario, Canada

We do research to understand what is happening now and how that can have future ramifications. In acne scar repair, reporting patient satisfaction in a standardized manner can achieve these goals. Dermatologic procedures for acne scars can be life-changing for the patient, but the extent of that depends on the patient satisfaction with results of the procedure. Until our research was completed, it was unknown how often patient satisfaction was reported in studies of these procedural interventions and how this was measured. In this concise article, we share our findings.

We had previously identified, in a systematic review, studies on patient-reported outcome measures regarding satisfaction with acne treatment in general.^1^ We adhered to the required standards for systematic search and quality assessment using a prespecified protocol and searched multiple databases from inception to June 2020 without language restriction. We found that beyond the studies on medical treatment of acne, there was a substantial number on treatment of acne scars. Of the 188 studies we identified reporting patient satisfaction with acne treatment, 97 (52%) were on procedural treatment of acne scars, even more than those on treating acne (91/48%). This shows that dermatological surgeons considered patient satisfaction a relevant and important outcome to measure. This increasing recognition was reflected in the number of studies reporting treatment satisfaction of acne interventions over time: 5 in 2001 to 2005, 20 in 2006 to 2010, 24 in 2011 to 2015, and 42 in 2016 to 2020.

Patients with acne scars can present with multiple acne scar morphologies—hypertrophic and atrophic (further differentiated into ice pick, saucer, rolling, and boxcar)—and potentially varying locations including the face and torso. Advances in dermatologic technology have provided a range of repair options for scar correction. This real-world spectrum is also reflected in our review, as we found the following procedures used in publications on acne scars: various laser and light devices (59), fillers (11), radiofrequency/focused ultrasound (10), microneedling (9), topicals/peels (7), subcision (7), and a combination of treatments (15). Laser treatments were mainly versions of CO_2_, erbium, or Yttrium aluminium garnet lasers. An overview of all the studies can be found in our original systematic review.^1^

**Figure 1.**
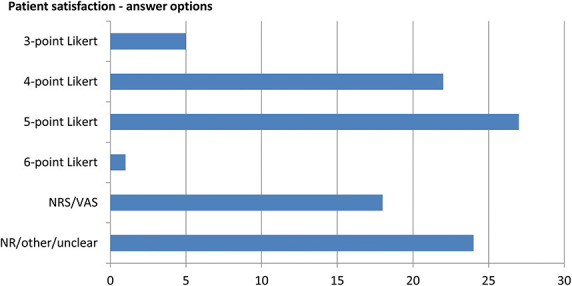
Overview of answer options for patients' treatment satisfaction questions. NR, not reported; NRS, numeric rating scale, 0 to 10 or 1 to 10; VAS, visual analog scale, 0 to 10 or 0 to 100.

We found great diversity in how patient satisfaction with these procedures was measured. Some researchers created their own questionnaire (27/27.8%), the majority asked one question on treatment satisfaction (58/59.8%), and 12.4% of the studies did not report the measure. Phraseology of questions and the response ranges differed even more. Some used an analog or a numeric rating scale, others a Likert scale from very dissatisfied to very satisfied (or the other way around). Please see Figure 1.

In itself, this diversity is not a problem. It is commendable that dermatologic surgeons help patients, conduct research, measure relevant domains, and share results by publishing. These efforts do provide very helpful information on a particular procedure or on the difference between the procedures that are being compared in a study. However, this diversity in reporting treatment satisfaction impedes future comparisons of patient-reported treatment satisfaction between studies and synthesis of results in meta-analyses.

The concept of satisfaction is implicit when evaluating the value of a treatment—to both the doctor and the patient. The importance of this concept has previously been identified by acne patients and by dermatologic surgeons as implied by our findings.^2^ Thus, on the critical domain of treatment satisfaction, it would be beneficial for dermatological surgeons to find common ground.

This need not be a laborious exercise unlike development of a globally accepted core outcome set for pharmacological interventions in acne. In addition to other relevant outcomes in their studies, if dermatological surgeons could agree on a single question on treatment satisfaction with the same response range, it would be both effective (to facilitate comparative assessments) and efficient (to reduce waste in resources). Within the scope of our findings, the singular question could be: “How satisfied are you with the result of the procedure?” and a corresponding 5-point Likert response scale (completely satisfied to completely dissatisfied). This standardization can help us understand the value of what we do now and can assist colleagues, researchers, and patients in future studies.
